# Combined Effect of pH and Neutralizing Solution Molarity on the Rheological Properties of Chitosan Hydrogels for Biomedical Applications

**DOI:** 10.3390/gels11030212

**Published:** 2025-03-18

**Authors:** Sofia Jansen de Medeiros Alves, Matheus Araújo Santos, João Emídio da Silva Neto, Henrique Nunes da Silva, Milena C. S. Barbosa, Marcus Vinicius Lia Fook, Rômulo Feitosa Navarro, Suédina Maria de Lima Silva

**Affiliations:** Northeast Biomaterials Evaluation and Development Laboratory (CERTBIO), Graduate Program in Materials Science and Engineering (PPG-CEMat), Academic Unit of Materials Engineering, Federal University of Campina Grande, Av. Aprígio Veloso, 882-Bodocongó, Campina Grande 58429-900, PB, Brazil; sofia.jansen@estudante.ufcg.edu.br (S.J.d.M.A.); matheus.santos@certbio.ufcg.edu.br (M.A.S.); joaoemidio2@gmail.com (J.E.d.S.N.); henrique.nunes@certbio.ufcg.edu.br (H.N.d.S.); milena.costa@certbio.ufcg.edu.br (M.C.S.B.); marcus.liafook@certbio.ufcg.edu.br (M.V.L.F.)

**Keywords:** oscillatory behavior, physically crosslinked hydrogels, physicochemical properties, shear behavior

## Abstract

Hydrogels are promising materials for biomedical applications due to their tunable properties. Despite significant research on optimizing the mechanical and rheological properties of chitosan hydrogels, a comprehensive analysis incorporating pH and molarity of the neutralizing solution is still lacking. This study addresses this gap by evaluating how these factors influence the rheological characteristics of chitosan hydrogels. The hydrogels were prepared using an acidic blend and were neutralized with sodium hydroxide solutions. Rheological characterization demonstrated that all samples exhibited pseudoplastic behavior, with viscosity decreasing under shear stress. Hydrogels with higher pH values exhibited lower viscosity, which is attributed to the reduced protonation and weaker electrostatic repulsion between chitosan chains. In contrast, more acidic conditions resulted in increased viscosity and greater chain entanglements. NaOH concentration impacted gel stability; lower concentrations resulted in more stable gels, whereas higher concentrations increased crosslinking but compromised integrity at elevated pH. These findings provide essential insights for optimizing chitosan hydrogels with customized properties, making them highly suitable for specific biomedical applications, such as advanced 3D-printed wound dressings.

## 1. Introduction

Hydrogels are a highly versatile class of materials, consisting of three-dimensional networks of hydrophilic polymers capable of absorbing and retaining large amounts of water or biological fluids. This unique absorption capacity makes hydrogels widely used in various biomedical applications, including controlled drug release, tissue engineering, and wound dressings [1,2,3]. Hydrogels can be classified into physical and chemical types, based on the polymer network formation mechanism. Chemically crosslinked hydrogels, characterized by covalent bonds between polymer chains, offer greater mechanical stability compared to physically crosslinked ones due to differences in the nature of the interactions that hold the polymer network together [4]. However, the potential for toxic by-products in chemically crosslinked hydrogels can limit their biomedical use, as they may generate adverse effects in living organisms even at low concentrations [5]. This risk is minimized in physically crosslinked hydrogels, which eliminate the need for chemical crosslinking agents and instead rely on reversible molecular interactions such as hydrogen bonding, hydrophobic and ionic interactions, and polymer chain entanglement [6]. These interactions allow physical hydrogels to respond to external stimuli, such as pH and temperature variations, a highly advantageous feature for biomedical applications [7,8,9].

Among the various polymers used in hydrogel formulation, natural polymers like chitosan have gained prominence due to their abundance, excellent biocompatibility, and biomimetic properties, positioning them as promising candidates for biomedical applications [10]. Chitosan, a derivative of chitin, consists of randomly distributed β-(1−4)-linked D-glucosamine and N-acetyl-D-glucosamine units [11,12,13]. It is the only naturally occurring cationic polyelectrolyte, extensively studied for its biocompatibility, biodegradability, and antimicrobial properties [13,14]. These characteristics make chitosan particularly suitable for the production of physical hydrogels, especially those that respond to pH and ionic strength changes, allowing for versatile applications in medicine and pharmaceutical sciences [15,16].

Despite the numerous advantages of chitosan hydrogels, optimizing their rheological properties for specific applications remains challenging. Properties, such as viscosity and structural stability, directly influence hydrogel performance under physiological conditions and their suitability for use in injectable systems or load-bearing tissues. Studies indicate that factors like chitosan concentration, system pH, and neutralizing solution molarity significantly affect the viscoelastic behavior of hydrogels [17,18]; however, the combined effects of these parameters have not yet been fully characterized in the literature. This gap in understanding limits the targeted development of chitosan hydrogels with optimized rheological properties for specific applications such as bioprinting that requires a hydrogel that possesses viscosity suitable for the injection process and post-printing dimensional stability.

To address this gap, the present study aims to investigate the influence of gel pH and the molarity of the neutralizing solution on the rheological properties of chitosan hydrogels. By analyzing their behavior under shear stress and oscillatory frequency sweep, this study seeks to provide a detailed rheological profile that can support the rational design of chitosan hydrogels with enhanced mechanical and functional properties for biomedical applications, more specifically bioprinting.

## 2. Results and Discussion

### 2.1. Organoleptic Properties and FTIR of Chitosan Hydrogels

**Figure 1:**
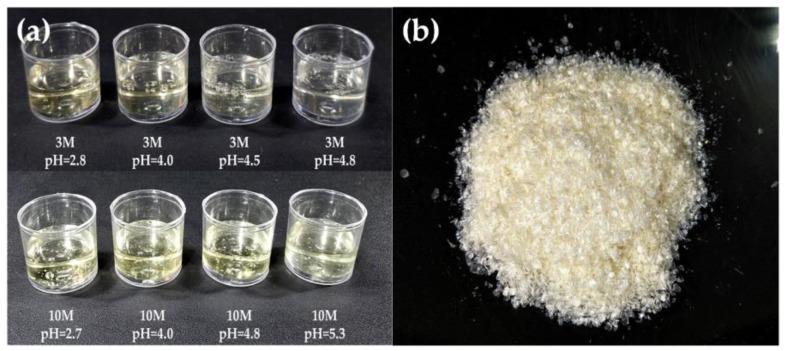
Chitosan hydrogels with different pH values (**a**) and CS powder (**b**).

Figure 1a shows the chitosan hydrogels, which exhibit a yellowish, translucent appearance, a viscous texture, and a characteristic vinegary odor. In contrast, the powdered form of chitosan (Figure 1b) appears light beige and is odorless. The pH of the hydrogels was adjusted using 3M and 10M NaOH solutions, which influenced their organoleptic properties. Hydrogels neutralized with the 10M NaOH solution demonstrated more consistent gelation and a milder odor compared to those treated with the 3M solution. The higher alkalinity of the 10M NaOH enabled more effective neutralization, resulting in a more stable hydrogel matrix with reduced residual acidity.

According to the data presented in Table 1, hydrogels neutralized with the 10M NaOH solution required significantly lower volumes of solution compared to those neutralized with 3M NaOH to achieve the same pH level. This efficient pH adjustment allowed the hydrogels to reach the desired pH range, which closely matches the natural pH of the skin (4.7–5.75). Maintaining this pH range is critical for the intended application, as this study aims to develop hydrogels suitable for 3D-printed wound dressings.

**Figure 2:**
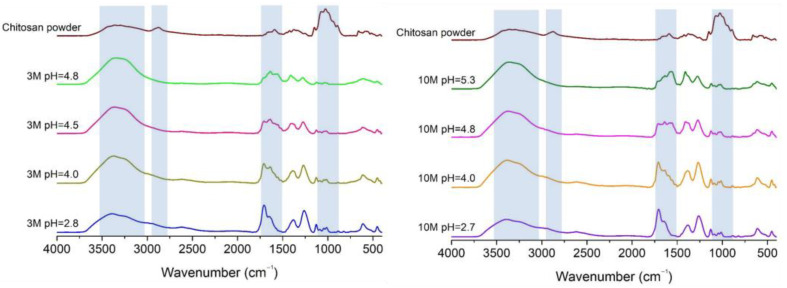
FTIR spectra of chitosan powder and chitosan hydrogel samples under different pH conditions, neutralized in 3M and 10M NaOH solutions.

Figure 2 presents the FTIR spectra of chitosan powder and chitosan hydrogel samples under different pH conditions, neutralized in 3M and 10M NaOH solutions. The observed spectral region around 3500–3200 cm^−^^1^ corresponds to the stretching vibrations of O-H and N-H bonds [19]. Its increase in intensity in the FTIR spectrum is due to the dissolution of chitosan powder in acidic solution and is likely attributable to the appearance of more protonated groups (-NH_3_^^+^^ and -OH), increasing the hydrogen bonding interactions in the polymer with neighboring molecules, intensifying the O-H stretching region [20,21,22]. It is possible to observe that the less intense peak, disregarding the spectrum of the chitosan powder, is related to pH 2.7 and 2.8, respectively, indicating the most acidic hydrogel samples. This behavior can be justified due to the intense presence of protonated groups that are not forming hydrogen bonds yet. However, with the addition of NaOH to the hydrogel samples, the pH values increase, leading to stronger hydrogen bonding interactions. This results in a more intense and broader peak at this spectral region [23].

The peak around 2900 cm^−^^1^, which is typically associated with the C-H stretching vibrations from aliphatic groups [24], practically disappears with the dissolution of chitosan in acidic solutions, regardless of the pH values of the samples. The enhanced hydrogen bonding network resulting, as explained previously, from the increased -NH_3_^+^ and -OH groups under acidic conditions can limit the visibility of C-H stretching vibrations at the spectrum. This strong network can decrease the independent vibrational freedom of C-H groups, which reduces the intensity of the C-H stretching peak at 2900 cm^−^^1^. According to Delmar and Bianco-Peled [25], the formation of hydrogen bonds lowers the frequencies of stretching vibrations and increases the frequencies of bending vibrations.

Peaks around 1650–1500 cm^−^^1^ are attributed to the amide I and amide II bands, which are associated with C=O stretching and N-H bending vibrations in the chitosan structure [26]. The chitosan powder presents a weak peak in this spectral region, indicating that it has a high degree of deacetylation [27]. The peak around 1560 cm^−^^1^, associated with amide II, becomes more pronounced after dissolving chitosan in an acidic solution, with greater intensity at lower pH values. This suggests that the amino group in the polymer is in its protonated -NH_3_^^+^^ form, acquired after acid dissolution, and that this cationic form is present in greater volumes at lower pH values [20,28].

The region around 1000 cm^−^^1^ typically corresponds to the C-O stretching and C-O-C linkage vibrations in the polysaccharide backbone of chitosan [29,30]. Similarly to the case of the peak around 2900 cm^−^^1^, the peak observed around 1000 cm^−^^1^ has a decrease in its intensity after the dissolution of chitosan in an acidic medium. As previously discussed, the acidic medium can protonate the amino groups of chitosan, enhancing hydrogen bonding interactions due to the increased number of protonated groups. These interactions can affect the vibrational freedom of C-O and C-O-C linkages, often resulting in broader and less intense peaks in this region [25], as shown in Figure 1. It is possible to observe that, with the addition of NaOH in the samples, the shapes, intensities, and positions of the peaks around 1000 cm^−^^1^ were basically the same, revealing that the chemical structures related to the C-O and C-O-C linkages of the hydrogels did not change when the medium’s pH was increased, corroborating the results of Yan et al. [31].

### 2.2. Rheological Properties of Chitosan Hydrogels

#### 2.2.1. Shear Behavior

**Figure 3:**
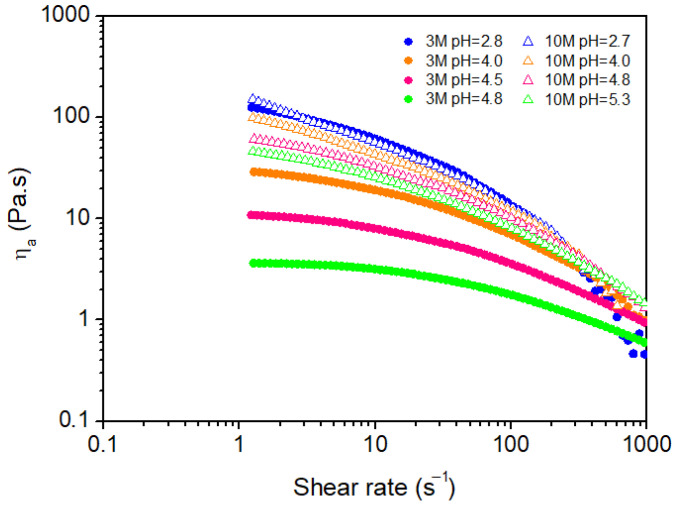
Flow behavior of the chitosan hydrogels.

Figure 3 illustrates the relationship between shear rate ($\dot{\gamma )}$ and apparent viscosity (for chitosan hydrogels under different pH conditions, using neutralizing solutions of varying molarities (3M and 10M).

All hydrogels displayed pseudoplastic (shear-thinning) behavior, evidenced by a reduction in viscosity with increasing shear rate. None of the hydrogels demonstrated yield stress, indicating that a sol–gel transition was not achieved within the applied shear rate range (1–960 s^−1^). This suggests that the hydrogels remained in a liquid-like state without forming a solid-like network, even at higher shear rates. Such non-Newtonian behavior implies that the hydrogel structure can reorganize under shear to facilitate flow, a characteristic advantageous for biomedical applications requiring injectability or controlled drug release.

The results further indicate that, at the same molarity of the neutralizing solution, hydrogels with higher pH values (i.e., those formed closer to neutral pH) display lower viscosities. This trend can be explained by the reduction in positive charges on chitosan chains as the pH approaches the pKa of chitosan (approximately 6.5). As Kang et al. [32] noted, lower protonation levels reduce electrostatic repulsion between polymer chains, creating a more flexible and less viscous hydrogel network. Conversely, hydrogels prepared at lower pH values (more acidic conditions) exhibit higher viscosities due to increased protonation of the chitosan chains. The resulting higher positive charge density along the polymer chains amplifies electrostatic repulsion, causing the chains to stretch and expand their hydrodynamic volume, thereby increasing overall viscosity [32,33].

Furthermore, as the chitosan chains extend under acidic conditions, the likelihood of chain entanglement rises. These physical entanglements hinder the free flow of the solution, making it more resistant to deformation and further increasing its viscosity [34]. Thus, the more stretched and extended the polymer chains are, the higher the probability of entanglements, which contributes to the gel-like consistency observed in chitosan solutions at lower pH. This phenomenon highlights how pH modulation can effectively tailor the rheological properties of chitosan hydrogels.

The stability of chitosan hydrogels as influenced by NaOH concentration was examined by evaluating the power-law index (*n*) in response to pH adjustment via NaOH solutions of different molarities (3M and 10M). Some fluids that do not exhibit Newtonian behavior may exhibit a relationship between shear stress (τ) and shear rate ($\dot{\gamma }$), represented by a power function, known as Ostwald’s Power-Law, given by the following equation:

(1) $$\tau \left(\dot{\gamma }\right)=K{\dot{\gamma }}^{n}$$

where *K* is the consistence index and n is the power-law index. The power-law index measures the departure from Newtonian behavior for which *n* = 1.0. For 0 < n < 1.0, the fluid will exhibit pseudoplastic behavior and its apparent viscosity will be reduced by increasing shear rate. For n > 1.0, the fluid will be dilatant and characterized by increasing apparent viscosity with increasing shear rate. If we apply the Napierian logarithm on both sides of Equation (1), we have

(2) $$\ln\tau \left(\dot{\gamma }\right)=n\ln\dot{\gamma }+\ln K$$

If Equation (2) represents a straight line, $n\neq f\left(\dot{\gamma }\right).$ Then, one can calculate n:

(3) $$n=\frac{d\ln\tau }{d\ln\dot{\gamma }}$$

**Figure 4:**
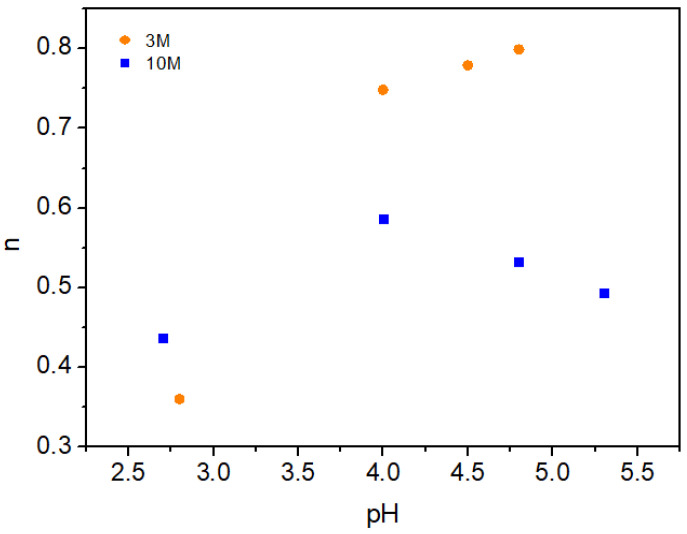
Variation of the power-law index (*n*) with pH for each molarity.

As shown in Figure 4, the behavior of the power-law index varied notably between hydrogels treated with 3M and 10M NaOH solutions, reflecting the significant impact of NaOH concentration on gel stability.

For the 3M NaOH solution, an upward trend was observed in the power-law index (*n*) as pH increased, suggesting enhanced stability with higher pH levels (i.e., toward neutral pH). This pattern indicates that at a moderate NaOH concentration, the deprotonation of chitosan’s amino groups contributes to more stable gel structures. This increased stability may be attributed to a favorable balance between deprotonation and electrostatic interactions, enabling stronger hydrogen bonding and ionic interactions within the hydrogel network.

In contrast, hydrogels treated with the 10M NaOH solution displayed a different trend. Initially, there was a slight increase in the power-law index (*n*) with pH; however, beyond a certain point, the index *n* began to decline with further pH increase. This behavior suggests that at higher NaOH concentrations, excessive deprotonation could reduce electrostatic stabilization among chitosan chains, potentially disrupting the gel network. As a result, the index *n* remained near an average value of 0.51, showing limited variability in comparison to the 3M-treated samples. This stability plateau could be beneficial for applications where a consistent gel structure is required, as excessive pH variability may compromise the gel’s integrity.

Overall, the findings indicate that moderate NaOH concentrations (3M) facilitate the formation of chitosan hydrogels with enhanced stability, characterized by an index *n* approaching unity. Meanwhile, higher NaOH concentrations (10M) yield a more uniform but potentially less resilient gel structure. This suggests that 10M NaOH treatment may be more appropriate for applications requiring consistent mechanical properties, while 3M NaOH could be advantageous for uses where higher stability is critical. These insights are essential for optimizing chitosan hydrogel preparation for diverse biomedical and industrial applications.

**Figure 5:**
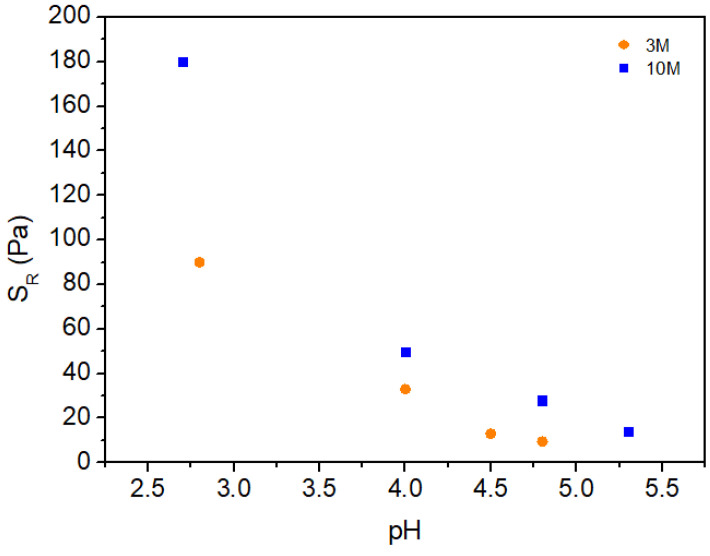
Variation of rupture stress (S_R_) with pH for each molarity.

The ionic strength of the neutralizing solutions (3M and 10M NaOH) plays a critical role in defining the properties of chitosan hydrogels, as demonstrated in this study. The higher ionic strength of the 10M NaOH solution increases the Na^+^ ion concentration, which influences electrostatic interactions, hydrogen bonding, and network density. This effect is observed in the FTIR spectra, where hydrogels treated with 10M NaOH exhibit more intense and broader peaks, suggesting stronger hydrogen bonding interactions and a denser hydrogel matrix. However, excessive ionic strength can compromise hydrogel integrity, as indicated by the reduced gel rupture stress (*S_R_*) and lower mechanical stability under shear stress (Figure 5).

**Figure 6:**
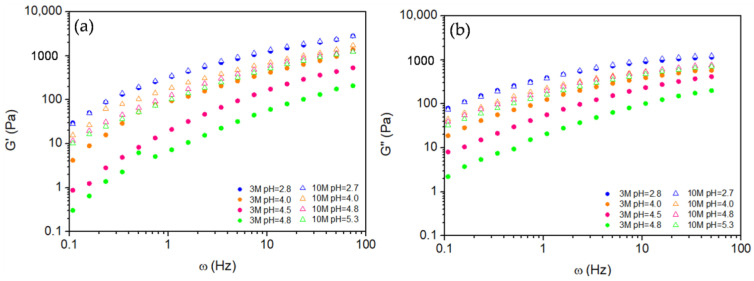
Frequency sweep for storage, G′(ω) (**a**), and loss, G″ (ω) (**b**), moduli.

In contrast, the lower ionic strength of the 3M NaOH solution provides a gentler neutralization process. This approach helps preserve electrostatic interactions among chitosan chains, contributing to a more stable hydrogel with improved structural integrity and viscosity under shear stress. The oscillatory behavior analysis (Figure 6) also indicates that hydrogels treated with 3M NaOH maintain higher stability at lower pH values, while those treated with 10M NaOH show a plateau effect in storage modulus (*G’*) at higher pH levels, suggesting a more uniform but potentially less resilient network.

These findings highlight that both pH and ionic strength are critical parameters for tailoring the physicochemical and mechanical properties of chitosan hydrogels. This is particularly important for applications such as 3D-printed wound dressings, where balancing stability and adaptability is essential for achieving the desired performance.

Figure 5 shows the behavior of the gel rupture stress (*S_R_*) as a function of pH for chitosan hydrogels modified with 3M and 10M NaOH solutions. In this study, we introduce the concept of gel rupture stress (*S_R_*) as the maximum stress at the onset of the initial Newtonian plateau in the hydrogel’s apparent viscosity, observed as a function of shear rate. It can be observed that *S_R_* decreases as pH increases for both NaOH concentrations, suggesting a reduction in the structural strength of the hydrogel with higher pH in the modifying solution. This effect occurs in both concentrations but is more pronounced in hydrogels modified with 10M NaOH solution, indicating a direct relationship between solution molarity and hydrogel stability under shear conditions.

Analyzing the results, it can be inferred that the higher molarity (10M) leads to more complete deprotonation of the amino groups in chitosan, negatively impacting the hydrogel’s structural integrity. This observation aligns with the hypothesis that at higher concentrations, NaOH promotes not only total deprotonation of the amino groups but also hydrolysis of bonds within the polymer structure, resulting in dissolution and loss of mechanical strength.

In contrast, hydrogels prepared with 3M NaOH exhibit a more gradual decrease in S_R_, suggesting that partial deprotonation of amino groups allows for ionic interactions between polymer chains, contributing to greater structural stability. Thus, the lower NaOH concentration results in hydrogels with better shear resistance, as the integrity of the glycosidic bonds is preserved, allowing for the formation of a more robust structural network.

These results demonstrate that the concentration of the NaOH solution plays a critical role in determining the mechanical stability of chitosan hydrogels. At lower concentrations, it is possible to obtain more stable hydrogels, likely due to the maintenance of ionic interactions and minimized degradation of the polymer structure. Conversely, higher concentrations result in a more pronounced weakening of the hydrogel, leading to reduced shear resistance.

#### 2.2.2. Oscillatory Behavior

Figure 6 shows the storage (*G′*) and the loss (*G″*) moduli as a function of frequency (*ω*) for chitosan hydrogels with pH modified using 3M and 10M NaOH solutions at various pH values. Both present the same pattern of variation with frequency for each pH and molarity considered, and except for the 3M sample at pH = 2.8 and the 10M sample at pH = 2.7, whose curves nearly coincide, all hydrogels modified by 10M NaOH exhibit higher *G′* and G″ values compared to their 3M counterparts across the entire frequency range. Given that the objective of the oscillatory experiment is to identify the maximum value of the storage modulus, *G′_P_*, we will focus on the evaluation of *G′*(*ω*).

The observed increase in G′ with higher molarity can be attributed to the greater availability of polymer chains for crosslinking, forming a more interconnected network. A denser network structure enhances the solid-like properties of the hydrogel, making it stiffer and more resistant to deformation. However, as pH increases, G′ decreases for NaOH concentrations, suggesting that higher pH values reduce the solid character of the hydrogel. This reduction in modulus with increasing pH means that it weakens the network density and reduces crosslinking, resulting in a softer, more flexible hydrogel structure.

At higher pH, chitosan deprotonation reduces electrostatic repulsion among polymer chains, enabling increased chain interactions and potential crosslinking. However, excessive deprotonation may also weaken hydrogen bonding between chitosan chains, ultimately reducing G′. This dual effect suggests that while controlled deprotonation can improve rigidity, excessive deprotonation disrupts intermolecular bonds critical to the hydrogel’s structural integrity.

These findings highlight the nuanced impact of NaOH molarity and pH on the mechanical properties of chitosan hydrogels. Higher molarity leads to stiffer hydrogels due to enhanced crosslinking, while higher pH levels reduce rigidity by decreasing both chitosan concentration and hydrogen bonding. This balance between crosslinking and deprotonation is essential for tailoring hydrogel properties for specific applications.

**Figure 7:**
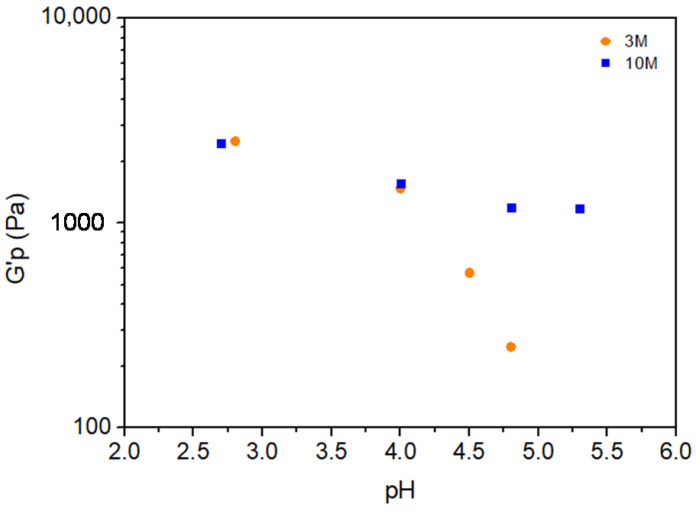
Variation in plateau storage modulus estimated from Figure 4 data with pH for each molarity.

Figure 7 illustrates the plateau storage modulus (G′_P_) as a function of pH for chitosan hydrogels modified with 3M and 10M NaOH solutions. The trend observed for G′_P_ closely parallels that of the gel rupture stress (S_R_) seen in Figure 4, confirming that the mechanical stability of the hydrogels decreases as pH increases. This correlation reinforces the notion that a higher pH reduces the network integrity of the hydrogel, leading to diminished mechanical properties.

However, an interesting distinction emerges between the two NaOH molarities. For pH values below 4.0, the G′_P_ values for hydrogels modified with both 3M and 10M NaOH remain nearly identical, indicating minimal influence of NaOH concentration on modulus in this pH range. This convergence suggests that, at lower pH values, both NaOH concentrations similarly affect chitosan deprotonation and crosslinking density, resulting in comparable mechanical stability.

Above pH = 4.0, the behavior of G′_P_ diverges based on the NaOH molarity. The 3M-modified hydrogel exhibits a continued decline in G′_P_ as pH increases, indicating a steady reduction in mechanical strength likely due to further chitosan deprotonation and network loosening. In contrast, the 10M-modified hydrogel shows a plateau in G′_P_, suggesting that higher NaOH molarity provides a stabilizing effect at elevated pH levels. This stability may be due to a more robust initial crosslinking density, which resists further structural degradation even at higher pH.

These findings underscore the critical role of NaOH molarity and pH in tuning the mechanical properties of chitosan hydrogels. While both 3M and 10M NaOH solutions yield similar modulus values at low pH, higher molarity imparts greater resilience against structural weakening at elevated pH, offering insights into optimizing hydrogel formulations for specific mechanical stability requirements.

**Figure 8:**
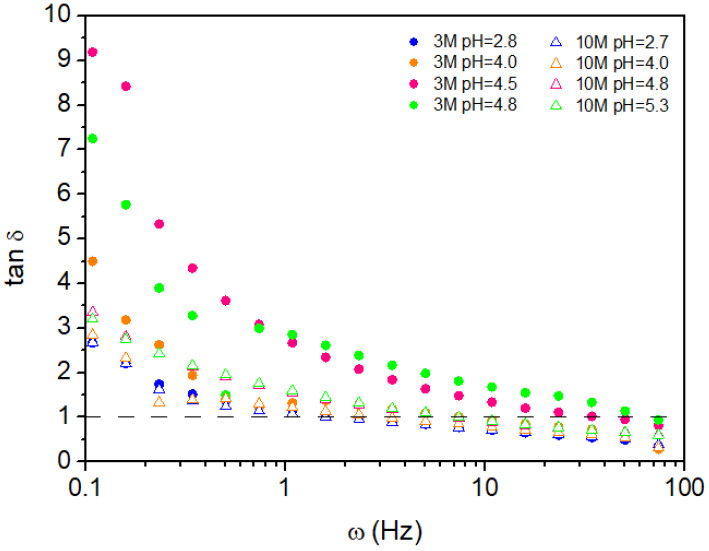
Frequency sweep for loss tangent, tan δ.

**Figure 9:**
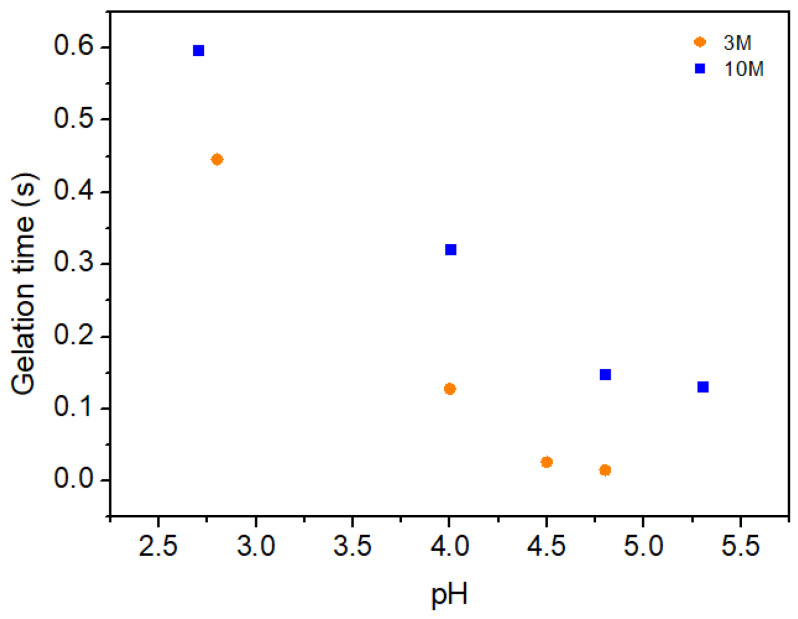
Variation in gelation time, t_g_, with pH for each molarity.

The results, illustrated in Figure 8 and Figure 9, provide comprehensive insights into the impact of pH modification and NaOH concentration on the viscoelastic properties and gelation dynamics of the hydrogels. Figure 8 demonstrates the variation in the loss tangent (tan δ) with angular frequency (ω) for all hydrogel samples. At lower frequencies, up to approximately 5 Hz, all samples exhibit a viscoelastic liquid-like behavior (tan δ > 1.0). This indicates that, under slow oscillatory deformation, the hydrogels primarily dissipate energy, displaying less solid-like elasticity. Notably, hydrogels derived from the 3M NaOH solution consistently exhibit higher tan δ values compared to those modified with the 10M NaOH solution across the entire frequency spectrum. This indicates a pronounced decrease in the elastic component of the 3M hydrogels, suggesting a less crosslinked or weaker network structure.

The 10M NaOH-modified hydrogels exhibit a markedly more elastic character than the 3M samples. This behavior can be attributed to the higher ionic strength and increased chitosan chain interactions facilitated by the more concentrated NaOH solution, leading to a denser and more robust hydrogel network. As the frequency increases, tan δ values converge closer to 1.0, signifying an increase in solid-like behavior, particularly for the 10M samples.

The relationship between gelation time and pH is presented in Figure 9. Here, it is evident that samples modified with the 10M NaOH solution exhibit longer gelation times compared to those prepared with 3M NaOH. The inverse correlation between crossover frequency (indicating the transition from liquid-like to solid-like behavior) and gelation time explains this phenomenon: as pH increases, the 10M samples maintain a more prolonged liquid-like state before reaching the gel point. This prolonged gelation time suggests that the network formation in the 10M-modified hydrogels is more gradual, likely due to stronger chain interactions and a higher degree of entanglement facilitated by the increased NaOH concentration.

The observed viscoelastic behavior and gelation dynamics highlight the critical role of both pH and NaOH molarity in determining the mechanical properties of chitosan hydrogels. The reduction in tan δ with increasing frequency, especially for the 10M samples, suggests that a higher NaOH concentration enhances the hydrogel’s ability to store and recover elastic energy, implying a more solid-like structure. In contrast, the 3M samples exhibit more liquid-like behavior, indicating a less densely crosslinked network.

These results highlight the influence of NaOH concentration and pH on the viscoelastic properties and gelation behavior of chitosan hydrogels. Higher NaOH molarity enhances the elastic character and structural integrity of the hydrogel, while pH modulates gelation kinetics and viscoelastic response. These findings offer critical insights for designing and optimizing chitosan-based hydrogels tailored to applications requiring specific mechanical and gelation properties.

**Figure 10:**
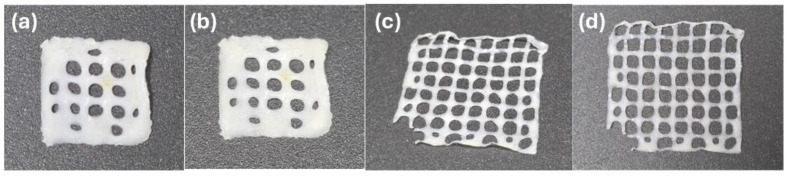
Variation in (**a**,**b**) 3D printed dressings with fewer pores per unit area and (**c**,**d**) with more pores per unit area.

Furthermore, our investigation into the effects of gel pH and neutralizing solution molarity provides a comprehensive rheological profile, enabling the development of chitosan hydrogels with enhanced properties for biomedical applications. The potential of these hydrogels is demonstrated in Figure 10, which showcases 3D-printed dressings with tailored pore densities to address specific clinical requirements. Panels (a) and (b) illustrate dressings with lower pore densities, while (c) and (d) present higher pore densities, underscoring the versatility of the 639 KDa chitosan for 3D printing applications.

## 3. Conclusions

This study aimed to investigate the influence of pH and the molarity of the neutralizing solution on the rheological properties of chitosan hydrogels, with a focus on developing materials suitable for biomedical applications. A preliminary study of 3D printing suggests application of these gels to 3D-printed wound dressings. Using a comprehensive methodology, chitosan hydrogels were formulated under varying conditions and characterized through advanced rheological and spectroscopic analyses. The key findings revealed that chitosan hydrogels exhibit pseudoplastic (shear-thinning) behavior, with viscosity and structural stability significantly affected by pH and neutralizing solution molarity. Hydrogels formed under more acidic conditions showed higher viscosities due to increased protonation and electrostatic repulsion, while higher pH conditions reduced viscosity and increased network flexibility. Moreover, moderate molarity (3M NaOH) produced more stable and resilient hydrogels, whereas higher molarity (10M NaOH) led to uniform but structurally weaker networks. Oscillatory tests demonstrated that higher NaOH concentrations enhanced the elastic modulus, resulting in a denser network, though structural weakening occurred at higher pH values. The rheological and mechanical properties of chitosan hydrogels can be finely tuned by adjusting these parameters, a critical feature for optimizing hydrogels for biomedical applications like bioprinting, where precise control over mechanical and viscoelastic properties is essential.

## 4. Materials and Methods

### 4.1. Materials

Chitosan (CS) with a molecular weight of 639 kDa and a degree of deacetylation of 96% was supplied by the Northeastern Biomaterials Evaluation and Development Laboratory-Certbio (Campina Grande, Brazil). This specific chitosan grade was selected after a comprehensive rheological evaluation of multiple samples (247 KDa, 270 KDa, and 400 KDa), which were previously investigated for applications such as hemostatic powders [35], self-healing materials [36], and dermal creams [37]. While these chitosan grades exhibited excellent properties for their respective applications, none demonstrated the ideal viscoelasticity and printability required for 3D printing. In contrast, the 639 KDa chitosan uniquely met these criteria, making it the optimal choice for optimizing 3D-printed wound dressings. Glacial acetic acid (CH_3_COOH, analytical grade—P.A.) and citric acid (C_6_H_8_O_7_.H_2_O, analytical grade—P.A.) were purchased from Química Moderna (Barueri, Brazil). Lactic acid 85% (C_3_H_6_O_3_, analytical grade—P.A.) and sodium hydroxide micropearls (NaOH) were acquired from Neon (Suzano, Brazil).

### 4.2. Preparation of Chitosan Hydrogels

The chitosan hydrogels were prepared following an adapted method from Wu et al. [38]. An acidic solution (100 mL) containing 0.12% (*w*/*v*) citric acid, 19.4% (*v*/*v*) lactic acid, and 38.8% (*v*/*v*) acetic acid was prepared for chitosan dissolution. This solution was obtained by mixing 97% (97 mL) of aqueous solution 1, prepared with 40% (*v*/*v*) acetic acid and 20% (*v*/*v*) lactic acid, with 3% (3 mL) of aqueous solution 2, prepared with 4% (*w*/*v*) citric acid. Chitosan was dissolved in the resulting acidic mixture at a concentration of 5% (*w*/*v*) under continuous stirring using a Fisatom 713DS mechanical stirrer (Fisatom, model 713DS, Fisatom Equipamentos Científicos Ltda, São Paulo, Brazil) at room temperature (25 °C) for 4 h to ensure complete homogeneity. The combination of acids was chosen to achieve a hydrogel with relatively high viscosity, a critical requirement for the 3D printing of microstructured and stretchable chitosan hydrogels. This study aims to develop a hydrogel suitable for 3D-printed wound dressings, where post-printing dimensional stability is significantly enhanced by using more viscous hydrogels.

**Figure 11:**
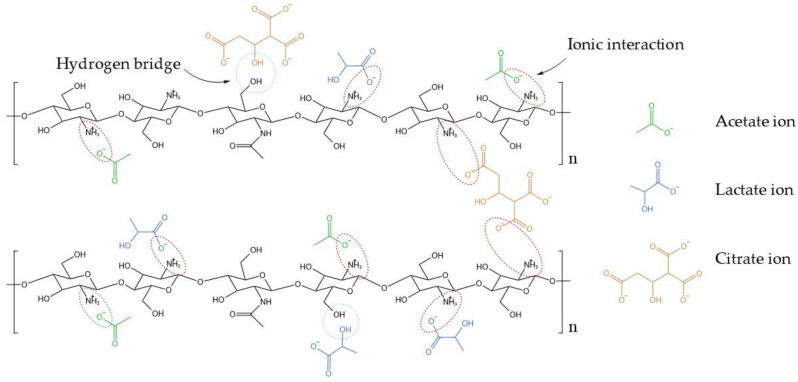
Molecular interactions in protonated chitosan hydrogel. Schematic representation of ionic crosslinking points formed between protonated chitosan chains and ionized citric, lactic, and acetic acids. These interactions stabilize the hydrogel network, enhancing rheological properties critical for 3D bioprinting applications.

The protonated chitosan structure and its crosslinking interactions with ionized acids are depicted in Figure 11.

For pH adjustment, the method described by Richa [39] was employed. Sodium hydroxide (NaOH) solutions at concentrations of 3 M and 10 M were gradually added to the hydrogel under continuous mechanical stirring to achieve samples with varying pH levels, tailored for rheological and structural property analyses. During the NaOH addition, the pH was meticulously monitored using an electronic pH meter (LiTH Ferramentas, model LT2404, LiTH Ferramentas, São Paulo, Brazil) to ensure precise alignment with the target pH values. The use of concentrated NaOH solutions was necessary because less concentrated solutions would require excessively large volumes to reach the target pH of approximately 5.0, which falls within the natural pH range of the skin (4.7–5.75). This pH range is crucial for the intended application, as this study focuses on developing a hydrogel suitable for 3D-printed wound dressings.

Table 1 presents the average volumes of the NaOH solutions (3 M and 10 M) required to adjust the pH of 1 g of chitosan hydrogel to the desired values. The data highlight the relationship between NaOH concentration, target pH, and the volume of solution added, providing insights into the pH adjustment process for further rheological and structural analyses.

To eliminate bubbles and prevent density inconsistencies that could interfere with rheological measurements, the hydrogel samples were transferred to 10 mL syringes and centrifuged using a Novatecnica NT 835 Benchtop Macro Refrigerated Centrifuge (Novatecnica, São Paulo, Brazil) at 2000 rpm for 15 min. This procedure ensured the preparation of uniform, bubble-free samples, which were then ready for subsequent analysis.

### 4.3. Characterization

Chitosan hydrogels were characterized to investigate their rheological and physicochemical properties, with particular attention to flow behavior and pH responsiveness. This study primarily focused on rheological characterization. Fourier transform infrared (FTIR) spectroscopy was employed to evaluate the chemical characteristics of the hydrogels, specifically assessing how pH influences their chemical structure. Spectra were recorded across a range of 650 to 4000 cm^−1^, using a Spectrum 400 FT Mid-IR spectrometer with ATR assembly with diamond crystal (PerkinElmer, Springfield, IL, USA), with each sample scanned 16 times at a resolution of 4 cm^−1^.

Rheological characterization was conducted using a HAAKE™ MARS™ rheometer (Thermo Fisher Scientific, Waltham, MA, USA) with a PP35TiL rotor in plate–plate configuration (0.5 mm gap). Frequency sweeps (0.1–100 Hz) were performed at a controlled temperature of 37 °C, alongside steady shear tests to evaluate viscosity as a function of shear rate, ranging from 1 to 960 s^−1^. Prior to measurement, the plate temperature was stabilized at 37 °C, and analyses began only after reaching temperature and strain equilibrium. These measurements provide critical insights into the viscoelastic properties and flow characteristics of chitosan hydrogels, underscoring their potential utility in diverse biomedical applications.

The rupture stress, *S_R,_* was defined in this work as the stress from which the apparent viscosity leaves its initial constant value, $\eta_{0}$, and begins the process of decreasing with the shear stress until a new equilibrium is reached, $\eta_{\infty }$. That is,

$$0\;\leq \tau <S_{R},\;\eta \left(\tau \right)=\eta_{0\;}\;and\;\frac{d\;\eta (\tau )}{d\tau }=0$$

$$\tau \geq S_{R},\;\eta =\eta (\tau )$$

And, *S_R_* is calculated by the modified Cross model presented in Equation (4):

(4) $$\eta (\tau )=\frac{\eta_{0\;}}{\left(1+{\left(\frac{\tau }{S_{R}}\right)}^{1-n}\right)}$$

The gelation time, $t_{g},\;is\;the\;inverse\;of\;angular\;frequency,\;\omega ,\;where\tan\delta =\frac{G^{\prime \prime }}{G^{\prime }}=1.0$, where *G′* is the storage modulus and *G″* is the loss modulus, that is

(5) $$t_{g}={\left(\frac{1}{\omega }\right)}_{\omega \;at\;\tan\delta =1.0}$$

## Figures and Tables

**Table 1:** Average volumes of NaOH solutions (3M and 10M) added per gram of the chitosan hydrogel to achieve specific pH values.

NaOH Solution Concentration	pH	Volume (mL) of NaOH Solution per g of Chitosan
3M	2.8	0
3M	4.0	5.38
3M	4.5	4.69
3M	4.8	6.92
10M	2.7	0
10M	4.0	2.00
10M	4.8	2.80
10M	5.3	2.59

## Data Availability

The raw data supporting the conclusions of this article will be made available by the authors upon request.
